# Toxicity of microplastics and nanoplastics: invisible killers of female fertility and offspring health

**DOI:** 10.3389/fphys.2023.1254886

**Published:** 2023-08-28

**Authors:** Yuli Geng, Zhuo Liu, Runan Hu, Yanjing Huang, Fan Li, Wenwen Ma, Xiao Wu, Haoxu Dong, Kunkun Song, Xiaohu Xu, Zhuo Zhang, Yufan Song

**Affiliations:** ^1^ Institute of Integrated Traditional Chinese and Western Medicine, Tongji Hospital, Tongji Medical College, Huazhong University of Science and Technology, Wuhan, China; ^2^ Department of Traditional Chinese Medicine, Tongji Hospital, Tongji Medical College, Huazhong University of Science and Technology, Wuhan, China; ^3^ Department of Neurosurgery, Tongji Hospital, Tongji Medical College, Huazhong University of Science and Technology, Wuhan, China

**Keywords:** microplastics, nanoplastics, reproductive toxicity, cross-generational, environmental toxicants

## Abstract

Microplastics (MPs) and nanoplastics (NPs) are emergent pollutants, which have sparked widespread concern. They can infiltrate the body via ingestion, inhalation, and cutaneous contact. As such, there is a general worry that MPs/NPs may have an impact on human health in addition to the environmental issues they engender. The threat of MPs/NPs to the liver, gastrointestinal system, and inflammatory levels have been thoroughly documented in the previous research. With the detection of MPs/NPs in fetal compartment and the prevalence of infertility, an increasing number of studies have put an emphasis on their reproductive toxicity in female. Moreover, MPs/NPs have the potential to interact with other contaminants, thus enhancing or diminishing the combined toxicity. This review summarizes the deleterious effects of MPs/NPs and co-exposure with other pollutants on female throughout the reproduction period of various species, spanning from reproductive failure to cross-generational developmental disorders in progenies. Although these impacts may not be directly extrapolated to humans, they do provide a framework for evaluating the potential mechanisms underlying the reproductive toxicity of MPs/NPs.

## 1 Introduction

**FIGURE 1:**
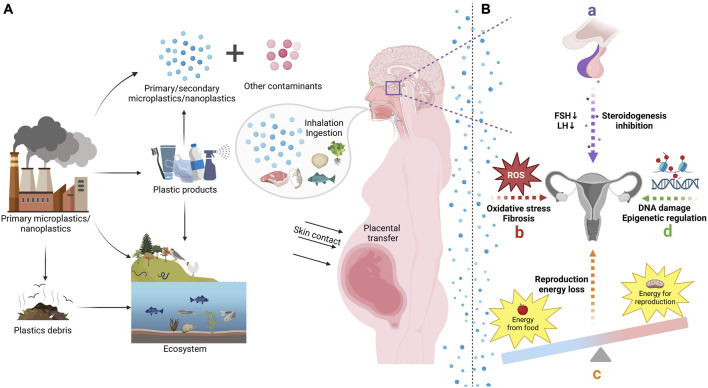
The sources, transfer, and reproductive toxicity of microplastics (MPs) and nanoplastics (NPs). **(A)** According to the different sources, MPs/NPs can be separated into primary and secondary type. Primary MPs/NPs have been generated and added to a range of daily necessities in order to meet business purposes. Plastic debris released into the environment will further degrade as secondary MPs/NPs. MPs/NPs can be transferred to human body through inhalation, ingestion, and skin contact, which pose a great threat to female reproduction and offspring health. **(B)** Exposure to MPs/NPs will interfere with reproductive endocrine in female, which will inhibit gonadotropic hormones and steroidogenesis. It will also cause a decline of energy allocated to reproduction and induce oxidative stress in female reproductive system. In addition, MPs/NPs may also affect female reproduction through DNA damage and epigenetic regulation.

With the intensification of industrialization, the world has now embraced an era of plastics. Polyethylene (PE), polypropylene, polyvinyl chloride, polystyrene (PS), polyurethane, and polyethylene terephthalate have hit 80% of plastic demand (Europe, 2015). The application of plastics has brought great convenience, but has also resulted in the discharge of a large quantity of plastic refuse into the environment, causing the accumulation of plastic in ecosystems (Figure 1) (Andrady and Neal, 2009). They can be found in a wide range of environments across the globe, including inland rivers, soil, air, and even polar regions (Ivar do Sul and Costa, 2014; Nor and Obbard, 2014; Anderson et al., 2016; Bessa et al., 2019). After entering the environment, bulk plastic materials will be broken down into small fragments by heat, photochemical reactions, oxidation, and other processes, thus forming microplastics that may persist for an extremely long period (Canniff and Hoang, 2018). In addition to environmental sources, plastics are produced in the form of microparticles and even incorporated into personal care items such as lotions, moisturizers, cleansers, and toothpaste to meet industrial requirements (Rist et al., 2018; Cox et al., 2019). When the size of plastic particles approaches the micron range, the interaction and absorption with organisms may become significant (Wright et al., 2013b). MPs are plastic particulates with diameters less than 5 mm, whereas NPs range in dimension from 1μm to 100 nm (Rochman et al., 2016). Recently, there has been growing concern regarding the fate and impact of MPs and NPs in the environment. MPs/NPs can be ingested and transmitted by animals, which may lead to toxicity in humans (Chae et al., 2018; Prokić et al., 2019; Strungaru et al., 2019). Previous research has demonstrated that MPs/NPs accumulate in a variety of organisms and have a wide range of negative consequences, including liver inflammation and intestinal flora disturbance (Jin et al., 2018; Yang et al., 2019). According to recent investigations, MPs/NPs have been detected in human hands and facial skin, hair, saliva, as well as placenta and feces (Schwabl et al., 2019; Abbasi and Turner, 2021; Ragusa et al., 2021; Xu et al., 2022). Additionally, numerous studies have revealed that MPs/NPs induce reproductive damage in different species and have extensive impacts on the developmental and metabolic abnormalities of offspring (Rist et al., 2017; Luo et al., 2019a; Liu et al., 2019b; Luo et al., 2019b; Jaikumar et al., 2019; Trifuoggi et al., 2019; Amereh et al., 2020; Park et al., 2020). Female reproductive disorder is a global health issue, which may be closely related to the environmental deterioration (Feichtinger, 1991; Mahalingaiah et al., 2016; Zhou et al., 2020). Moreover, given that pregnancy is a crucial period time for neonatal organ development, prenatal exposure to these toxins is of particular concern for the health and development of unborn child (Gómez-Roig et al., 2021; Yi et al., 2022). However, it remains unclear of the mechanisms that MPs/NPs entangle with female reproductivity. Therefore, this review summarizes the connections between MPs/NPs and female fertility, pregnancy as well as offspring, in order to give some inspiration for investigating the reasons of the high incidence of female infertility and enhancing the protection of female fertility and offspring health.

## 2 Endocrine interference of MPs/NPs

Some research has suggested that MPs/NPs may act as endocrine disruptors (Sussarellu et al., 2016; Wang et al., 2019). It has been demonstrated that exposure to MPs/NPs will affect the levels of sex hormone in serum of aquatic organisms and rodents (Wang et al., 2019; Feng et al., 2022; Wei et al., 2022). According to a study conducted on female marine medaka, MPs have a detrimental regulatory effect on the hypothalamic pituitary gonadal (HPG) axis, which is accompanied by a decline in gonadotropic hormones and suppression of steroidogenesis (Wang et al., 2019). In an experiment with swine granulosa cells, it was also confirmed that NPs exposure influenced steroidogenesis, especially the synthesis of estrogen and progesterone (Basini et al., 2021). However, there is a strong heterogeneity in the dynamic changes of female reproductive endocrine related hormones caused by MPs/NPs. In an experiment using aged polystyrene microplastics (PSMPs), it was found that PSMPs could stimulate the production and release of estradiol (E2) and increase the expression of estrogen receptor (Yang et al., 2022b). This is controversial in light of the results obtained with primary MPs, which may be due to the modifications in the properties of plastic particles (Wang et al., 2019). Through transcriptome analysis, Sussarellu et al. also verified the differential expression of hormone receptors or transcripts involved in different hormone pathways in oysters subjected to MPs (Sussarellu et al., 2016). In spite of this, they found that MPs contained endocrine disrupting compounds, indicating that additives in MPs might interfere with the experiment (Sussarellu et al., 2016).

According to the current research, we may not be able to draw a definite conclusion on how MPs/NPs affect the endocrine axis. Existing results continue to suggest that MPs/NPs may have endocrine-disrupting properties, yet further experiments are required to rule out the influence of plastic properties and additives.

## 3 Reproductive toxicity of MPs/NPs

### 3.1 Bioenergy utilization

Energy is essential for the growth of organisms, while ingestion of MPs/NPs may lead to digestive tract obstruction, reducing food intake and energy intake (Zhang W. et al., 2020). Different types of MPs/NPs exposure have been reported to limit female reproductive capacity in a variety of species, which were attributed to the restrictions on energy consumption (Au et al., 2015; Cole et al., 2015; Sussarellu et al., 2016; Cong et al., 2019; Kim et al., 2019; Mao et al., 2022). In the marine worm *Arenicola marina*, researchers have observed extended intestine residence time, inflammation, and depletion of energy reserves following the exposure to MPs, which may be related to reproductive issues (Wright et al., 2013a). Through transcriptome analysis, Sussarellu and colleagues also confirmed that the PS-MPs intake altered the energy flow and metabolism of oysters, resulting in a loss of energy allocated to the reproductive function (Sussarellu et al., 2016). Despite the fact that animals tend to increase their appetite in response to external stress, studies have demonstrated that exposure to MPs still has an influence on the metabolism of glucose and protein in female *Drosophila melanogaster* with an increased food consumption (Zhong et al., 2022). However, the reproductive influence of MPs/NPs on energy reserve differ among species. For zooplankton like *Daphnia magna*, green algae, which can colonize and flourish on the surface of plastic objects, is the primary food source (Gross et al., 2016). As a result, the presence of plastic materials can either impede or assist organisms by occupying intestinal space during plastic ingestion, such as supporting the growth of algae in the environment (Canniff and Hoang, 2018). Several studies have found that zooplankton exposed to high concentrations of plastic particles tended to produce more offspring (Ogonowski et al., 2016; Rist et al., 2017; Liu et al., 2019b; Eltemsah and Bøhn, 2019). Canniff et al. found that polyethylene MPs had no influence on the reproduction of *Daphnia Magna*, despite the digestive system being stuffed with microplastic beads (Canniff and Hoang, 2018). Whereas some studies have discovered a reduction in the number of newborns in the reproduction test employing MPs and NPs (Besseling et al., 2013; Zimmermann et al., 2020).

Currently, the controversy about the impact of MPs/NPs on energy metabolism by impairing or obstructing the digestive tract is primarily centered on zooplankton and other species, which may depend on the characteristics of the food, as mentioned above (Canniff and Hoang, 2018). The hypothesis that MPs/NPs induce reproductive toxicity by altering energy distribution seems credible for the majority of the investigated organisms. It is worth noting that the studied species are quite tiny, which is a significant factor for the impact of MPs/NPs on their digestive systems. However, extrapolating these findings to humans may be challenging. Although there is evidence that the digestion of microplastics may weaken intestinal barriers (Hirt and Body-Malapel, 2020), additional research is necessary to establish a connection between energy exhaustion and reproductive disorders.

### 3.2 Oxidative stress

MPs/NPs have been proved to exhibit pro-oxidant properties (Jeong et al., 2017; Trifuoggi et al., 2019; Dubey et al., 2022; Ferrante et al., 2022). The toxicity of MPs/NPs in organisms mainly comes from oxidative stress through the generation of reactive oxygen species (ROS). The accumulating ROS then triggers a sequence of biological responses, such as oxidative stress-induced signaling cascades, apoptosis and inflammation (Jeong et al., 2017). Numerous studies have demonstrated that the activation of oxidative stress *in vivo* may be connected to the detrimental effects of MPs/NPs on the reproduction of various species (Jeong et al., 2016; Kim et al., 2019; Qiang and Cheng, 2021).

When *Paracyclopina nana* was exposed to MPs, researchers observed an increase of ROS levels which was related to impaired reproductive function with the decrease of newborn nauplii (Jeong et al., 2017). They also demonstrated an activation of mitogen-activated protein kinase/nuclear factor erythroid 2-related factor 2 (MAPK/Nrf2) signaling pathway, promoting the activity of antioxidant enzymes in response to the oxidative stress (Jeong et al., 2017). Additionally, they showed that the toxicity was inversely proportional to the size of MPs (Jeong et al., 2017). Some other investigations supported the association between MPs/NPs-induced oxidative stress and reduced fertility (Liu et al., 2019b; Trifuoggi et al., 2019; Qiang and Cheng, 2021; Xue et al., 2021). They have also shown an enhanced expression of genes encoding antioxidant enzymes to withstand environmental stress (Liu et al., 2019b). However, Liu et al. further found that the high concentration of NPs was likely to disrupt the antioxidant system in *Daphnia pulex*, manifesting as a reduction in the expression of the genes for antioxidant enzymes (Liu et al., 2019b).

In addition, the toxic effects of MPs/NPs have been validated in rodents. Research has revealed that MPs can induce oxidative stress and impair the antioxidant capacity in the ovary (Wei et al., 2022). As a consequence of PSMPs exposure, MPs were found to deposit in the ovary, which further resulted in decreased ovarian reserve, lower ovarian volume, and disruption of the estrous cycle (An et al., 2021; Hou et al., 2021; Feng et al., 2022; Haddadi et al., 2022; Liu et al., 2022; Wei et al., 2022). Wei et al. reported that PS-MPs reached the mouse ovary after oral administration of 5 μm fluorescent PSMPs for 2 days (Hou et al., 2021). In ovarian tissue, ROS and malondialdehyde (MDA) levels increased markedly whereas glutathione (GSH) levels decreased considerably (Hou et al., 2021; Wei et al., 2022). Hou et al. found that MP-induced oxidative stress further activated NOD-like receptor thermal protein domain associated protein 3 (NLRP3)/Caspase-1 signaling pathway, which led to pyroptosis and apoptosis of granulosa cells (An et al., 2021; Hou et al., 2021). Moreover, research by An et al. suggested that the activation of oxidative stress caused by PSMPs might also play a role in the ovarian fibrosis through the Wnt/β-catenin signaling pathway, as it could be the blocked by N-Acetyl-l-cysteine (NAC) treatment (An et al., 2021). Additionally, exposure to PSMPs altered the expression of cytoskeleton protein in rat ovary which was also considered as the target of ROS (Haddadi et al., 2022).

Apart from ovary, PSMPs were also observed to accumulate in the uterus, causing pathological alterations such as thinner endometrium, a reduction in uterine glands, endometrial adhesion and so on (Haddadi et al., 2022; Liu et al., 2022). Wu et al. claimed that via the Toll-like receptor 4/NADPH oxidase 2 (TLR4/NOX2) signaling axis PSMPs induced oxidative stress, which thereby activated Notch and transforming growth factor-β (TGF-β) signaling pathways, ultimately resulting in uterine fibrosis in rats. Inhibition of TLR4/NOX2 signaling transduction can play an anti-fibrotic effect by lowering the generation of ROS (Wu et al., 2022).

## 4 Genotoxicity of MPs/NPs

### 4.1 Deoxyribonucleic acid (DNA) damage

Currently, it has been documented that MPs/NPs may induce genotoxicity through DNA damage in various tissues and organs of several species (Zheng et al., 2019; Domenech et al., 2021; Sangkham et al., 2022; Guimarães et al., 2023). Two separate studies revealed that the reproductive toxicity induced by PSMPs may be associated with the activation of cell apoptosis through DNA damage in *Caenorhabditis elegans* (Chen et al., 2022; Hua et al., 2023). Wu and colleagues observed an increase in DNA damage markers in ovarian granulosa cells following exposure to PSMPs (Wu et al., 2023). Hua et al. also confirmed that suppressing DNA damage checkpoints could improve germline apoptosis and subfertility (Hua et al., 2023). An *in vitro* testing by Chatterjee et al. confirmed the toxicity of polystyrene nanoplastics (PSNPs) to zebrafish oocytes, accompanying an alteration in the expression of genes associated to DNA damage (Chatterjee et al., 2022).

### 4.2 Epigenetic response

Epigenetic regulation refers to the chemical modifications of DNA and histones that can influence gene expression without altering the DNA sequence (Skvortsova et al., 2018; Chen et al., 2020). Existing data on NPs and epigenetics suggest that NPs indeed have the potential to modulate the epigenome, while current research in this area remains limited. Wang et al. noticed that exposure to PSNPs led to a decrease in the expression of a methyltransferase, homoserine O-acetyltransferase (MET-2), which played a critical role in the germline cells in defending against the toxicity of PSNPs in *Caenorhabditis elegans* (Wang et al., 2021). Yang et al. also demonstrated that germline microRNA38 in *C. elegans* mediated epigenetic regulation in response to exposure to PSNPs (Yang et al., 2020). Additionally, MPs/NPs may cause cross-generational epigenetic effects since epigenetics is heritable, as will be discussed in the following section.

## 5 Cross-generational toxicity of MPs/NPs

**FIGURE 2:**
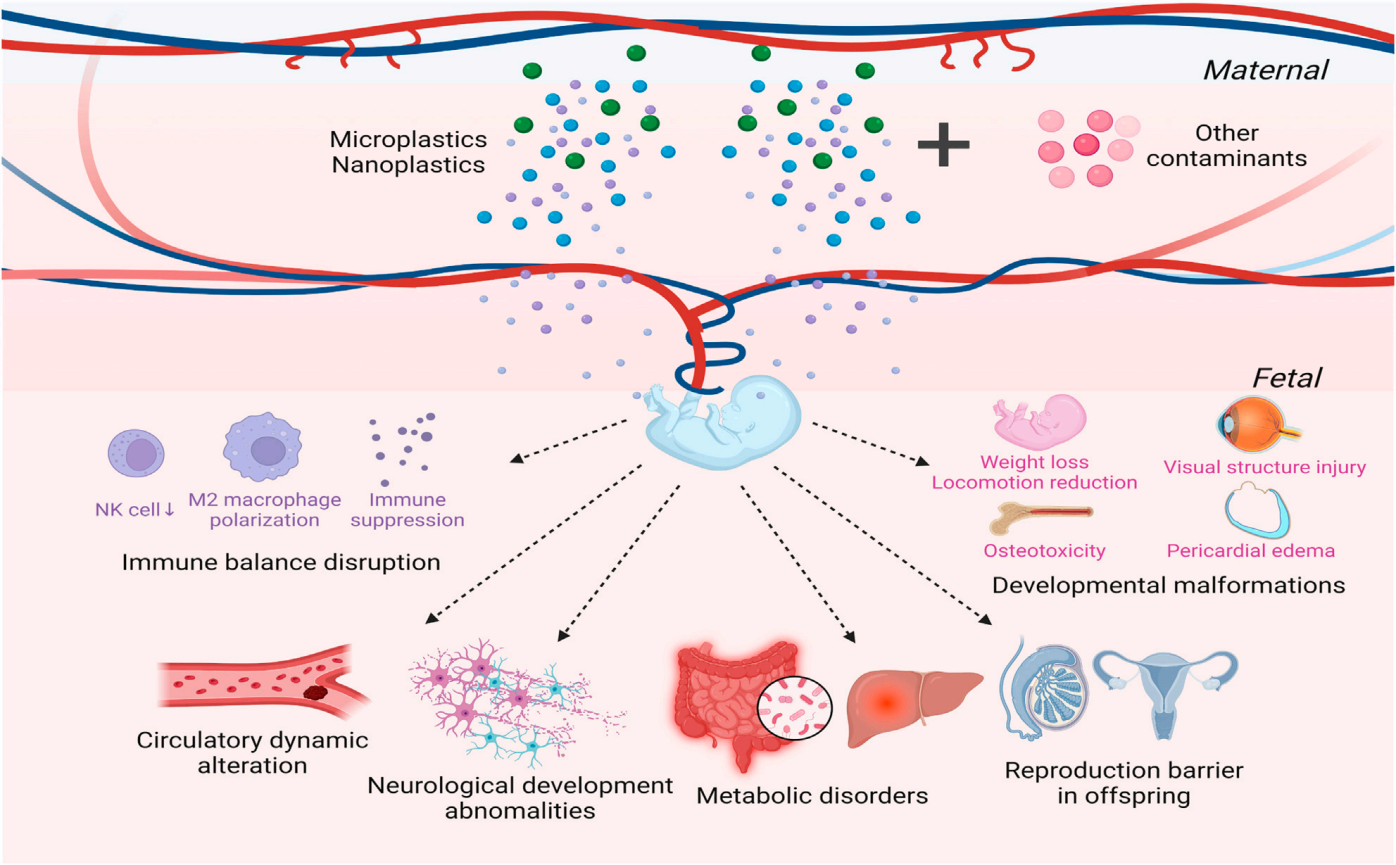
Cross-generational toxicity of microplastics (MPs) and nanoplastics (NPs). MPs/NPs have the potential to infiltrate the embryonic chorion. MPs/NPs will disrupt the delicate immune balance at the maternal-fetal surface while also altering the distribution and profile of immune cells in offspring. Additionally, MPs/NPs will impact fetal circulatory function and angiogenesis during pregnancy, inducing cardiovascular damage and hypercoagulable state. MPs/NPs will also cause neurological dysfunction, which can extend far beyond the gestational period. Moreover, metabolic disorders may emerge in progenies due to maternal exposure to MPs/NPs. Other detrimental influences of MPs/NPs manifest as developmental abnormalities and subfertility in progenies, leading to a multitude of health challenges throughout lifetimes.

MPs/NPs have the potential to translocate to oocytes, placenta, and offspring, thereby posing a greater threat to human and animal health (Figure 2). The cross-generational transfer effect of NPs has been illustrated in some aquatic and soil organisms (Manabe et al., 2011; Cui et al., 2017; Zhao et al., 2017; Pitt et al., 2018b; Teng et al., 2022). After exposing zebrafish to PSNPs, Pitt et al. found that PSNPs initially appeared in the yolk sacs of embryos that were maternally or co-parentally exposed rather than paternally exposed, and subsequently spread throughout the digestive system, pancreas, and liver in the larvae (Pitt et al., 2018b). Although it is unclear how maternal transfer of PSNPs is mediated, previous research has indicated that PSNPs interacted with vitellogenin, which may promote PSNPs transfer to the oocyte and eventually the embryonic yolk sac (Pitt et al., 2018b). Two independent research groups respectively demonstrated that PSNPs could penetrate the chorion of zebrafish and distribute to various organs after being absorbed via the yolk sac (Pitt et al., 2018a; Lee et al., 2019). Lee and colleagues described the morphology of chorion exposed to NPs with uneven surface as well as narrow chorionic pore canals under scanning electron microscope (Lee et al., 2019). NPs with sizes of 50 nm and 200 nm were dispersed throughout the pores, whereas NPs with a diameter of 500 nm were partially blocked, suggesting that the size of NPs was positively correlated with the capacity to penetrate (Lee et al., 2019). However, some experts still hold the view that chorion acts as an effective barrier against PSNPs. Their studies showed that PSNPs gathered on the chorion instead of infiltrating it (van Pomeren et al., 2017; Duan et al., 2020). Kashiwada et al. also verified that chorion could prevent embryos from PSNPs invasion at the early developmental stage of Japanese medaka. Nonetheless, with the extension of exposure time, it was found that NPs were internalized and transmitted to the gallbladder (Kashiwada, 2006). Researchers speculated that particles will not be transported across the chorion until the chorion reaches its maximal adsorption value (van Pomeren et al., 2017). Given the extent of chorionic pore canal in fish, most investigations supported that MPs will be blocked by embryonic chorion (LeMoine et al., 2018; Duan et al., 2020; Li et al., 2020; Cheng et al., 2021; De Marco et al., 2022). Additionally, exposure to NPs has also been shown to result in translocation from matrix to the placenta and fetal tissues in rodents (Huang et al., 2015; Fournier et al., 2020; Nie et al., 2021; Yang et al., 2022a). However, according to a single exposure experiment, carboxylated PSNPs only showed a high density in the placenta without reaching the embryonic tissue, and were eliminated within 4 days after exposure (Kenesei et al., 2016). Variations in exposure time and surface modifications of plastic particles may account for the discrepancies between different research. This work reminds us that the scavenging effect of MPs/NPs should be taken into consideration during exploration of their toxicity, which may have been overlooked in prior studies.

Unlike animal experiments, MPs have been detected in human meconium and placenta, including fetal side, maternal side, and chorionic amniotic membrane (Ragusa et al., 2021; Wei et al., 2022). Placental transfer of PSNPs from maternal to fetal compartment has been proven using the *ex vivo* human placental perfusion model (Wick et al., 2010; Grafmueller et al., 2015a). The findings of Grafmueller et al. suggested that an active, energy-dependent transport pathway rather than passive diffusion may be the underlying mechanism of PSNPs translocation across the placenta, in which syncytiotrophoblast played a crucial contributor (Grafmueller et al., 2015a). Although there are certain limitations in the methodology of human placental perfusion (Grafmueller et al., 2015b), these data are essential for comprehending the onset of developmental toxicity of MPs/NPs. Another *in vitro* study using human placental choriocarcinoma cells also verified the intercellular transport of PSNPs from the maternal to the fetal compartment, with an inverse relationship between particle size and transport rate (Cartwright et al., 2012).

Of note, a study pointed out that maternally administrated PSNPs were mainly transmitted to offspring through breast milk after delivery, while the quantity of PSNPs that crossed the placental barrier during pregnancy could not be enough to reach embryonic organs (Jeong et al., 2022).

There has been increased concern on the threat that MPs/NPs may pose to fetal development. Numerous studies have demonstrated that maternal exposure to MPs/NPs has profound influence on offspring at various levels, ranging from weight loss, developmental malformations to immune disturbance, metabolic disorders, circulatory abnormalities, neurological deficits, and reproductive failure (Luo et al., 2019a; Luo et al., 2019b; Bringer et al., 2020; Hu et al., 2021).

### 5.1 Immune microenvironment

Despite variations in exposure patterns, several investigations have elucidated that MPs/NPs induce embryo resorption (Fournier et al., 2020; Hu et al., 2021; Nie et al., 2021). After intraperitoneal exposure to PS-MPs, Hu et al. observed a reduction in uterine blood supply and the proportion of decidual natural killer (dNK) cells while an increase of placental T helper cells, a polarization of M2 macrophage, and an immune suppressive state of cytokines (Hu et al., 2021). Due to the intricacy of gestation, this result may not entirely align with our general conception (Yi et al., 2019). Therefore, further research is necessary to uncover the mechanism underlying the MPs/NPs induced embryonic loss. Additionally, a study reported that PE-MPs altered the composition of lymphocyte subsets in spleen of offspring which might be secondary to maternal and/or paternal toxicity (Park et al., 2020). Exposure of zebrafish embryos to MPs/NPs indicated that could trigger an immune response, which was also evidenced by the recruitment of neutrophils and macrophages around the PS particles (Veneman et al., 2017; Brun et al., 2018).

### 5.2 Circulatory dynamic

Although several of the studies previously cited lend credence to the notion that the embryonic chorion can effectively prevent external contaminants, it does not mean getting rid of the influence caused by MPs/NPs. The adsorption of MPs/NPs on the outer surface of chorion has been shown to alter the permeability of chorionic channel and the mechanical properties of embryonic chorion, which might result in the hypoxic microenvironment in the embryo (Duan et al., 2020; Cheng et al., 2021). Through the methodology of metabonomics, Duan et al. concluded that the variations in heart rates and blood flow rates were connected to the changes in the antioxidant system of the embryo (Duan et al., 2020). Furthermore, Park et al., reported that MPs/NPs caused pathological angiogenesis and peripheral microcirculatory disruption, thus leading to prematurity and growth restriction during the zebrafish embryonic development (Park and Kim, 2022). Sun and colleagues also provided evidence for NPs inducing cardiovascular damage in zebrafish embryos (Sun et al., 2021). They found that NPs can inhibited blood flow velocity of zebrafish embryos, resulting in hypercoagulable state of circulation (Sun et al., 2021). Simultaneously, NPs probably caused vascular cell dysfunction *in vivo* by inducing systemic inflammatory response and oxidative stress, which would eventually promote thrombosis in zebrafish embryos (Sun et al., 2021). The formation of atrioventricular heart valves was substantially impacted after human induced pluripotent stem cells were exposed to NPs, according to another *in vitro* investigation utilizing gene set enrichment analysis (Bojic et al., 2020).

### 5.3 Neurological development

According to multiple studies, prenatal or perinatal exposure to NPs may accumulate in fetal brain and result in brain dysfunction (Yang et al., 2022a; Jeong et al., 2022). A study injecting of PSNPs into zebrafish embryos also illuminated that NPs accumulated in the brain and induced oxidative DNA damage (Sökmen et al., 2020). Yang et al. illuminated that NPs induced excessive production of ROS, which led to apoptosis of fetal thalamic neurons and inhibition of γ-aminobutyric acid (GABA) synthesis, thus ultimately causing anxiety-like behavior in progenies (Yang et al., 2022a). Jeong et al. reported that PSNPs altered the composition of neural cells in the brain of postnatal offspring, featured as an increase in the number of astrocytes (Jeong et al., 2022). Furthermore, reductions of estrogen signal induced by PSNPs could lead to cognitive impairment in female offspring (Jeong et al., 2022). After embryo injection exposure, Zhang et al. described that there was a downregulation of genes involved in neurological function, including synapse formation, neuronal differentiation, and cytoskeleton modulation, which suggested an influence on the development of central nervous system (Zhang et al., 2020a). It was also confirmed by Nie et al. that exposure to NPs during the gastrula stage of chicken embryos caused neural tube defects (Nie et al., 2021). According to an investigation of Chen et al., embryo exposure to NPs lowered acetylcholinesterase (AChE) activity and substantially upregulated neurotoxicity biomarkers, which in turn affected the locomotion of juvenile fish (Chen et al., 2017).

### 5.4 Glucolipid metabolism

A team has revealed that maternal exposure during pregnancy to MPs would have cross-generational consequences, causing lipid and amino acid metabolic abnormalities in offspring, which might provide concealed risks for long-term metabolic diseases (Luo et al., 2019a; Luo et al., 2019b). This may be associated with gut microbiota dysbiosis and gut barrier dysfunction in matrix, according to their findings (Luo et al., 2019a). Another study found that prenatal and *postpartum* administration with PSNPs not only perturbed glucose metabolism but also triggered oxidative stress and inflammation in the liver of male offspring, thus resulting in a weight loss at birth and postnatally (Huang et al., 2022). Similarly, modifications in intestinal microbiota and glucolipid metabolism were noted after zebrafish embryos were exposed to MPs/NPs (Veneman et al., 2017; Wan et al., 2019).

### 5.5 Reproductive barrier in offspring

Diverse species have exhibited decreased fecundity in their progeny after parental exposed to MPs/NPs, and one study even discovered that *Daphnia Magna* required at least three generations to gradually recover from the effects of impaired fertility (Zhao et al., 2017; Martins and Guilhermino, 2018; Sobhani et al., 2021). Huang et al. revealed that exposure to PSNPs during pregnancy and lactation also prevented spermatogenesis in male offspring by causing testicular developmental disorders and oxidative injury (Huang et al., 2022). Additionally, Lu et al. reported that exposure to PSMPs altered the reproductive endocrine level of zebrafish embryos, exhibiting a considerable increase in testosterone, estrogen, vitellogenin, and T3 levels (Lu et al., 2022). Several studies also implied that the cross-generational reproductive toxicity was also associated with DNA methylation and histone modifications although one study found no significant differences in global DNA methylation among four generations in *Daphnia magna* (Yu et al., 2021; Song et al., 2022; Lee et al., 2023). These data offer fresh insight into the negative reproductive consequences of MPs/NPs on progeny which also require further investigations in the exact mechanisms.

### 5.6 Developmental malformations

A number of investigators have documented the aberrant development of progenies after parental exposure to MPs/NPs, even if some others have found no appreciable difference in the malformation rate of offspring (Chen et al., 2017; Pitt et al., 2018a). Body length reduction, weight loss, locomotion diminution, and changes in activity behavior are among the most typical developmental issues (Pacheco et al., 2018; Oliviero et al., 2019; Sun et al., 2019; Bringer et al., 2020; Bringer et al., 2022; Teng et al., 2022). Recently, a novel study evaluated the presence of MPs in fresh human placenta and the association with the development of neonatal infants, heightening concerns about the potential impact of a lifestyle involving continuous plastic exposure on birth outcomes (Amereh et al., 2022). Their research revealed a negative correlation between MPs burden and anthropometric measurements of neonates in intrauterine growth restriction (IUGR) pregnancies (Amereh et al., 2022). Additionally, MPs/NPs will induce pericardial edema and harm the integrity of the visual structure since they can reach heart, eyes, and other significant organs (Zhang et al., 2020b; Bojic et al., 2020; Sun et al., 2021; De Marco et al., 2022). Some studies also pointed out that MPs/NPs induced osteotoxicity which was inherited by offspring (De Marco et al., 2022; Tarasco et al., 2022).

## 6 Combined effects of MPs/NPs with other pollutants in reproductivity

MPs/NPs rarely play a solitary function in inducing biological toxicity in the natural environment, as evidenced by an abundance of recent literature (Xu et al., 2020; Sheng et al., 2021; Zhang et al., 2022). On the one hand, various types of additives are applied in the production of plastics to impart specific properties (Cole et al., 2011; Bouwmeester et al., 2015). In turn, these toxic additives will also leach out from MPs/NPs and cause deleterious effects (Bouwmeester et al., 2015; Sheng et al., 2021). Despite the fact that multiple studies have demonstrated that additives in plastics such as bisphenol A and phthalates can impair female reproductive function, research on the combined effects of MPs/NPs with these chemicals remains limited (Hunt et al., 2003; Lai et al., 2017; Li et al., 2018; Ma et al., 2019; Park et al., 2019; Ullah et al., 2022). On the other hand, due to their minuscule diameters and large specific surface area, MPs/NPs tend to absorb or desorb other environmental contaminants thus altering their bioaccumulation and toxicity (Law and Thompson, 2014; Yu et al., 2019). Several studies have raised concerns about the potential for MPs and NPs to serve as carriers for other environmental contaminants, triggering reproductive disorders such as endocrine disruption and infertility in females (Wu et al., 2023).

### 6.1 MPs/NPs and organic pollutants

Research on the interaction between MPs/NPs and organic pollutants implies that MPs/NPs have multiple effects on the bioaccumulation and toxicity of diverse compounds. A majority of studies have supported that the combined exposure of organic pollutants with MPs/NPs has an additive or synergistic toxicity on female reproduction and embryonic development. Endocrine disrupting chemicals (EDCs) are prevalent organic contaminants in the environment, which are likely to interplay with MPs/NPs (Liu et al., 2019a; Coffin et al., 2019; Hu et al., 2020; He et al., 2021; Lu et al., 2021). The co-occurrence of MPs/NPs and several EDCs has been reported to exerted an additive or synergistic endocrine-disrupting toxicity in reproductivity, impairing the ovarian function and inhibiting the secretion of sex hormones (He et al., 2021; Han et al., 2022; Mao et al., 2022; Lin et al., 2023a; Lin et al., 2023b). Furthermore, research indicated that PSMPs could enhance the desorption of di-(2-ethylhexyl) phthalic acid, thereby generating DNA oxidative damage, granulosa cell cycle arrest, and necroptosis in the ovary (Coffin et al., 2019; Wu et al., 2023).

Likewise, the comprehensive toxicity of MPs/NPs not only cause parental reproductive dysfunction, but also onset more notable cross-generational consequences in progenies. Compared to individual exposures, it has been confirmed that the combined exposure of MPs/NPs with multiple types of organic pollutants poses a more severe threat to offspring development, especially in cases of pericardial cyst, skeletal abnormalities, and growth retardation (Lu et al., 2022; Tarasco et al., 2022; Lin et al., 2023b; Gao et al., 2023; Zhang et al., 2023). On the basis of the combination index, Lu et al. also observed an antagonistic effect between MPs and sulfamethoxazol, while it only caused a slight reduction in the combined toxicity (Lu et al., 2022).

Even though most current studies suggest that MPs exacerbate the toxicity of organic pollutants, there is still controversy surrounding this issue. A study has indicated that there is no interaction between PSNPs and BPA in marine water medium, accompanying no alterations in the embryonic developmental toxicity on a phenotypic level (Ferrari et al., 2022). Some other studies pointed out that MPs/NPs could mitigate the toxicity of several compounds including B[α]P, phenanthrene, and butyl methoxydibenzoylmethane, lessening the adverse effects on embryonic development (Li et al., 2020; Liu et al., 2021; de Mello Souza et al., 2023). When zebrafish embryos were exposed to NPs and a mixture of complex polycyclic aromatic hydrocarbons (PAHs), PAHs were shown to adsorb onto the surface of NPs, thereby reducing developmental abnormalities and vascular injury (Trevisan et al., 2019). Additionally, a study found that NPs and phenmedipham (PHE) exhibited results of no interaction, synergistic effect, and antagonistic effect at different concentrations and endpoints (Santos et al., 2022). Despite that, dual exposure still increased the possibility of PHE transfer to embryos, disrupting oxidative balance and neural neurotransmitter activity (Zhang et al., 2023).

It is worth mentioning that most studies only focus on a specific type of pollutant, whereas the natural environment contains a vast array of compounds. Their coexistence may pose greater ecological or health hazards than their individual effects, necessitating additional investigation into these cumulative effects.

### 6.2 MPs/NPs and inorganic pollutants

Currently, investigations on the combined toxicity of MPs/NPs with inorganic substances predominantly focus on heavy metals (HMs) (Yan et al., 2020; Cheng et al., 2021; Santos et al., 2021; Feng et al., 2022). Yan et al. has found that the combined exposure to HMs and MPs led to the formation of empty follicles in marine medaka fish, showing additive and synergistic effects on reproductive toxicity (Yan et al., 2020). In addition, a study on co-exposure suggested that the presence of PSMPs increased the bioaccumulation of lead in mice, potentially exacerbating the ovarian toxicity through oxidative and endoplasmic reticulum stress in female mice (Feng et al., 2022). Santos et al. also found that MPs regulated neurotoxicity induced by copper in the early developmental stage of zebrafish, with higher AChE inhibition observed in the mixture groups (Santos et al., 2021). However, according to the research of Cheng et al., MPs could assimiliate HMs like cadmium present in the exposed environment, thereby reducing the bioaccumulation of HMs in embryos and performing a detoxifying function under co-exposure with MPs (Cheng et al., 2021). Besides, a study found that the combined effect of HMs and MPs can also influenced by the concentration of the mixture (Martins et al., 2022).

## 7 Discussion

Collectively, MPs/NPs tend to elicit multiple reproductive consequences in a variety of organisms, leading to the decline of female fertility and the developmental anomalies of offspring. However, it is still premature to make firm judgements regarding the toxicity on humans. On the one hand, several studies have demonstrated that the susceptibility of various species to MPs/NPs exposure varied with body size, showing a more severe toxicity with the decrease of body size (Jaikumar et al., 2019; Cormier et al., 2021). In comparison to the size of most experimental animals, MPs/NPs seem negligible to humans. However, for human beings, we are more likely to be confronted with MPs/NPs exposure over an extended period of time, or even throughout the entire life cycle, allowing for the accumulation of MPs/NPs in the organisms as well as the detrimental effects locally and systemically. On the other hand, the outcomes of laboratory research may not accurately represent the natural environment. The extent to which MPs/NPs could pose issues for different organisms will be determined by a variety of factors, such as particle size, the amount and type of plastic particles, the species-specific toxicity of plastics, the mode and location of plastics accumulation within organisms. Plastic debris exists in a diversity of shapes, sizes, and types in natural environments (Cole et al., 2011; Eerkes-Medrano et al., 2015; Syberg et al., 2015). Since most research adopted commercially available round primary MPs/NPs, it would be hard to completely replicate the exposure situation in nature. In addition, the concentration of microplastics in the environment could be less than 1 μg/L (Lenz et al., 2016). However, to better explore the toxic effects of MPs/NPs, most research has far exceeded the concentrations observed in the environment up to this point (Browne et al., 2008; von Moos et al., 2012; Besseling et al., 2013; Cole et al., 2015; Sussarellu et al., 2016). As we have seen in most investigations, the modest concentration of MPs/NPs may do no harm (Zhao et al., 2017; Eltemsah and Bøhn, 2019; Schöpfer et al., 2020). A study also demonstrated that cross-generational effects caused by environmentally relevant concentrations of PSMPs can be negligible or reversible (Qiang et al., 2020). Furthermore, a large number of studies have also revealed MPs/NPs can interact with multiple pollutants on account of their intense adsorption to other contaminants. As a result, it is still a highly complex issue how MPs and NPs endanger female reproduction and offspring development in the actual environment.

According to the current research, we still in support of MPs/NPs may play a significant role in female reproduction and have far-reaching influence beyond reproduction under certain conditions. We anticipate further mechanical research to shed light on the potential impacts of MPs/NPs with environment-related concentrations on female fertility and progeny health throughout the whole reproductive cycle.
